# TiO_2_ Hollow Spheres With Flower-Like SnO_2_ Shell as Anodes for Lithium-Ion Batteries

**DOI:** 10.3389/fchem.2021.660309

**Published:** 2021-12-08

**Authors:** Ying Weng, Ziying Zhang, Huizhen Zhang, Yangyang Zhou, Xiaona Zhao, Xingran Xu

**Affiliations:** ^1^School of Materials Engineering, Shanghai University of Engineering Science, Shanghai, China; ^2^School of Management, University of Shanghai for Science and Technology, Shanghai, China

**Keywords:** hollow spheres, tin dioxide, titanium dioxide, anodes, lithium-ion batteries

## Abstract

SnO_2_ is a promising anode material for lithium-ion batteries due to its high theoretical specific capacity and low operation voltage. However, its poor cycling performance hinders its commercial application. In order to improve the cycling stability of SnO_2_ electrodes, novel flower-like SnO_2_/TiO_2_ hollow spheres were prepared by facile hydrothermal method using carbon spheres as templates. Their flower-like shell and mesoporous structure highlighted a large specific surface area and excellent ion migration performance. Their TiO_2_ hollow sphere matrix and 2D SnO_2_ nano-flakes ensured good cycle stability. The electrochemical measurements indicated that novel flower-like SnO_2_/TiO_2_ hollow spheres delivered a high specific capacity, low irreversible capacity loss and superior rate performance. After 1,000 cycles at current densities of 200 mA g^−1^, the capacity of the flower-like SnO_2_/TiO_2_ hollow spheres was still maintained at 720 mAh g^−1^. Their rate capacity reached 486 mAh g^−1^ when the current densities gradually increase to 2,000 mA g^−1^.

## Introduction

As one of the most promising energy storage devices, rechargeable lithium ion batteries (LIBs) have been widely used in smart phones, computers, electric vehicles and other portable electronic devices due to their long cycle life and high energy density (Lee et al., 2009; Scrosati et al., 2011; Tian et al., 2017; Fan et al., 2019). However, graphite, as a commercial anode material for LIBs, has gradually lost its competitiveness in practical application given it poor rate property and low theoretical capacity (372 mAh g^−1^) (Li et al., 2014; Zhang J. et al., 2019). Compared with graphite, metal oxide anode materials have attracted much attention because of their high theoretical specific capacities (Chen et al., 2015; Jiang et al., 2019; Li et al., 2019; Liu et al., 2019; Zhang Z. et al., 2019).

Among metal oxides, tin dioxide (SnO_2_) was expected to be a promising anode material for LIBs given its high theoretical specific capacity (781 mAh g^−1^) and low operation voltage (Ao et al., 2020; Liu Q. et al., 2020). Unfortunately, SnO_2_ can not be applied as a LIB anode material alone due to the poor cycling performance resulted from the large volume variation during lithium ion insertion and extraction, resulting in crushing, and structural disintegration (Zhu et al., 2014; Cheong et al., 2017; Liu Q. et al., 2020). In order to improve the cycling stability of SnO_2_ electrodes, various nano-structured SnO_2_ materials have been developed (Yang et al., 2011; Ji et al., 2013; Jean et al., 2017; Mao et al., 2021; Shen et al., 2021). Among them, the hollow spheres were deemed to be an ideal structure of the anode materials for LIBs due to their low density, high surface-to-volume ratio, isotropic physical properties and structural stability (Miao et al., 2016; Wu et al., 2019). However, due to the limitation of dynamics, the pure SnO_2_ hollow spheres are still easy to be broken during the long period of charging and discharging, especially at a high current density (Tian et al., 2019). Composite with other materials was considered to be an effective way to alleviated the structural disintegration of SnO_2_ and improve its electrode cycling stability (Wu et al., 2015; Wu K. et al., 2018; Tian et al., 2019). In recent years, although the rate capacity of TiO_2_ is relatively low (~170 mAh g^−1^), TiO_2_ was widely recommended as the anode composite matrix material for lithium-ion batteries given its high operating voltage, low price, and less volume change during the lithium-ion insertion and extraction processes (Luo et al., 2016; Li et al., 2017; Tian et al., 2018; Xu et al., 2020). In addition, the reasonably arranged two-dimensional (2D) single-layer nano-flakes endow the electrodes good cycling stability due to their large effective contact area and high tolerance to volume variations (Wang et al., 2011; Fan et al., 2020). Therefore, the specific capacity and the cycling stability of SnO_2_-based electrode materials were expected to be further improved by vertical self-assembly of 2D SnO_2_ nano-flakes on the surface of TiO_2_ hollow spheres. However, how to vertically assemble 2D SnO_2_ nano-flakes on the surface of TiO_2_ hollow spheres to obtain uniform hollow composite spheres was still a major challenge given the aggregation characteristics of nanoparticles and their dependence on the synthetic environment.

In this study, three-dimensional (3D) hierarchical flower-like SnO_2_/TiO_2_ hollow composite spheres were prepared by a facile two-step hydrothermal synthesis. The flower-like SnO_2_ shell imparted these hierarchical hollow spheres a large specific surface area and high specific capacity. The TiO_2_ hollow matrix supported the composite products free from collapse during charging and discharging. Their hollow structure and the existence of 2D SnO_2_ nano-flakes made these hollow composite spheres more stable to cycle, even at high current density.

## Experiment Section

### Materials

All reagents are analytical grade and do not require further purification when used. Anhydrous Ethanol (CH_3_CH_2_OH > 99.8 %), tin chloride pentahydrate (SnCl_4_·5H_2_O), Titanium tetrachloride (TiCl_4_), anhydrous glucose (C_6_H_12_O_6_), sodium hydroxide(NaOH), hexadecyl trimethyl ammonium bromide (CTAB), acetic acid (CH_3_COOH), and sodium borohydride (NaBH_4_) were all purchased from Shanghai Sinopharm Chemical Reagent Co., Ltd.

### Synthesis of Carbon Sphere Templates

In a typical experiment, 89 g anhydrous glucose was dissolved in 250 mL deionized water and stirred at room temperature for 30 min. After stirring, the solution was transferred into a 100 mL Teflon-lined stainless steel autoclave. The autoclave was maintained at 180°C for 5 h, and then cooled to room temperature. After washing with deionized water and anhydrous ethanol, the products were dried at 80°C for 8 h. The black brown products obtained were the carbon sphere templates.

### Synthesis of TiO_2_ Hollow Microspheres

Carbon spheres were firstly synthesized by glucose hydrothermal method. 0.6 g carbon spheres were added to the prepared 30 mL titanium tetrachloride solution for ultrasonic dispersion for 15 min. The mixture was stirred at room temperature for 6 h, and then centrifuged and washed with acetic acid. The obtained products were placed in an oven at 80°C for 8 h. Then, they were annealed in air at 500°C for 3 h. After cooling to the room temperature, TiO_2_ hollow spheres were obtained.

### Synthesis of 3D Flower-Like SnO_2_/TiO_2_ Hollow Spheres

In a typical procedure, 0.5 g SnCl_4_·5H_2_O, 0.33 g NaOH, 0.74 g CTAB, and 30 mL deionized water were firstly mixed in a 50 mL glass tube reactor. Then 0.6 g the as-prepared TiO_2_ hollow microspheres were dipped into the mixed solution, dispersed by ultrasonic for 15 min, and stirred at room temperature for 6 h. The mixture was transferred to a 50 mL Teflon-lined autoclave and heated at 120°C for 12 h. The products were centrifuged and washed with deionized water and absolute ethanol for at least three times. After dried in an oven at 80°C for 6–10 h, they were annealed at 500°C for 3 h.

### Structure and Morphology Characterization

The structure of the obtained hollow composite spheres were determined by X-ray powder diffraction (XRD, Panalytical X' Pert, Holland) with Cu-Ka radiation (λ = 1.5418 Å). The morphologies of the obtained products were examined by Scanning Electron Microscopy (SEM, JSM-7000F, Japan) and Transmission Electron Microscopy (TEM, TitanX 60e300, USA). A Scanning Electron Energy-dispersive X-ray spectrometer (EDS) was attached to the TEM to analyze the composition of the specimens. The surface elemental electronic states and chemical bonds of materials were analyzed by X-ray photoelectron spectroscopy (XPS, PHI 5600 ESCA). The Brunauer-Emmett-Teller (BET) method was used to calculate the specific surface area of the products using nitrogen adsorption-desorption isotherm obtained by Micrometics Tristar 3000 system.

### Electrochemical Measurements

Using lithium-metal foil as the cathode, the CR 2032 coin cells were assembled in an argon-filled glove box with <0.5 ppm of water and oxygen. The cathode was made of a coating containing 80% active material, 10% super-P-Li carbon black and 10% polyvinylidene fluoride (PVDF). 1 M LiPF6 was dissolved in ethylene carbonate and diethyl carbonate with a volume ratio of 1:1 as electrolyte. The cells were galvanostatically charged and discharged on Neware-CT3008 battery tester. Cyclic voltammetry (CV) was conducted on a PARSTAT 4000 electrochemical workstation at a scan rate of 0.2 mV s^−1^ in the range of 3.0–0.01 V (vs. Li/Li^+^). Electrochemical impedance spectroscopy (EIS) was carried out in the frequency range of 100 kHz−0.01 Hz with an ac perturbation voltage of 5 mV.

## Results and Discussion

**Figure 1:**
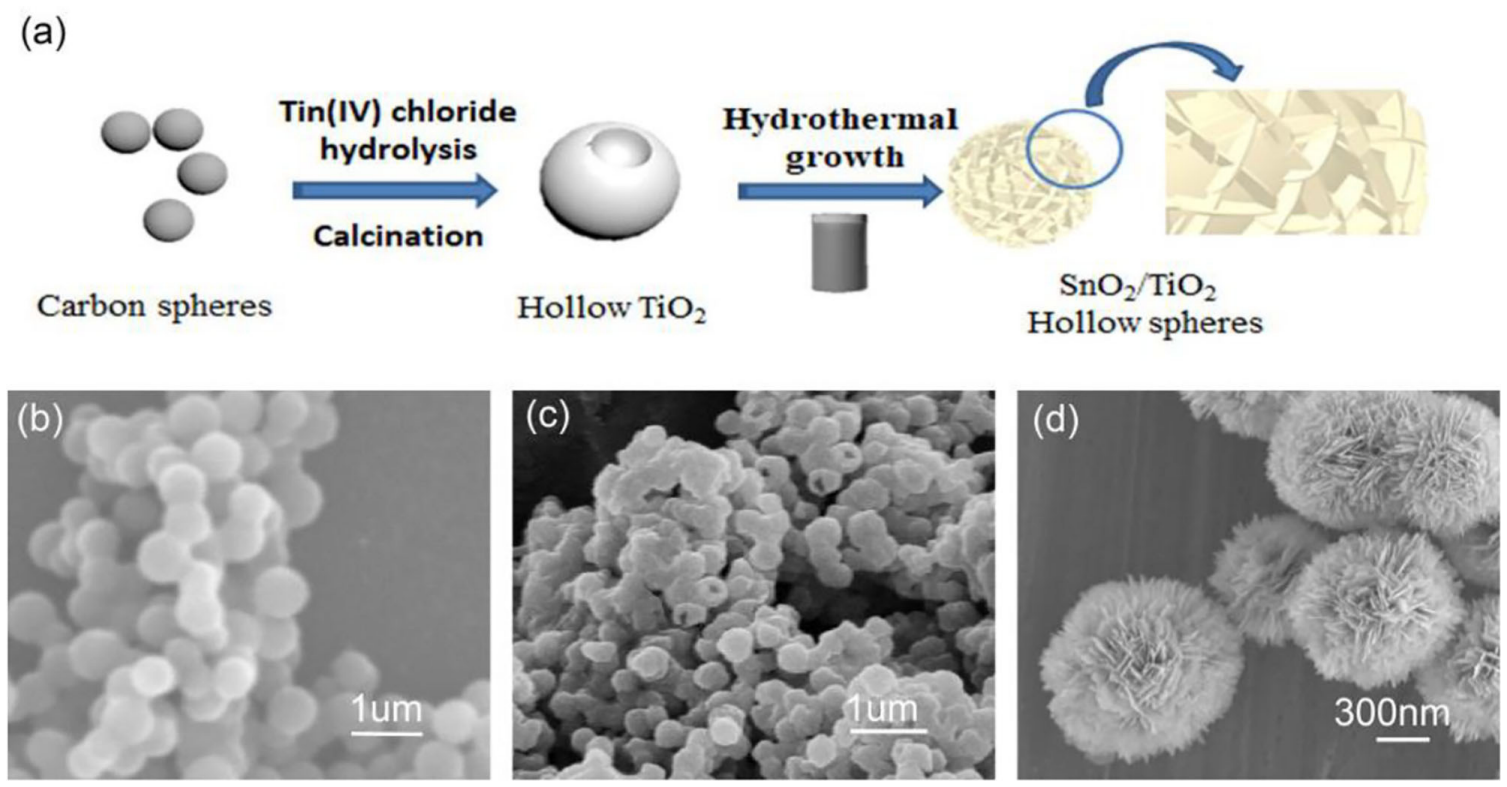
**(a)** Schematics of the fabrication process of 3D flower-like SnO_2_/TiO_2_ hollow spheres, **(b)** SEM image of the pure carbon spheres, **(c)** TiO_2_ hollow spheres, **(d)** 3D flower-like SnO_2_/TiO_2_ hollow spheres.

The synthesis process of 3D hierarchical flower-like SnO_2_/TiO_2_ hollow composite spheres is schematically illustrated in Figure 1a. TiO_2_ coatings were formed on the surface of the carbon spheres by direct hydrolysis of titanium tetrachloride. TiO_2_ hollow spheres were obtained by the calcination of the above hydrolyzed products. Subsequently, ultrathin SnO_2_ nano-flakes were assembled on the surface of TiO_2_ hollow spheres under hydrothermal conditions to form 3D hierarchical flower-like SnO_2_/TiO_2_ hollow composite spheres. Here, CTAB, as one of amphiphilic chemicals, not only acted as a dispersant, but also was easy to adhere to crystal surface due to its double layer structure (Qi et al., 2018). Therefore, with the help of CTAB, the SnO_2_ nanoparticles tend to grow along a specific crystallographic plane during the hydrothermal process, forming SnO_2_ nano-flakes.

Figure 1b shows the SEM images of the pure carbon spheres with smooth surface. The average diameter of carbon spheres was about 600 nm. The SEM image of the semi-closed spheres indicates that the TiO_2_ spheres clearly exhibited a hollow structure (Figure 1c). They were similar to carbon spheres in diameter. Figure 1d displays the SEM images of 3D hierarchical flower-like SnO_2_/TiO_2_ hollow composite spheres. The 2D SnO_2_ flakes were vertically assembled on the surface of the TiO_2_ hollow spheres to form a flower-like shell. The magnified image of the semi-closed spheres shows that the SnO_2_/TiO_2_ composite spheres presented a significant hollow architecture and their flower-like shell was assembled from SnO_2_ nano-flakes with a thickness of about 25 nm (Supplementary Figure 1a). This flower-like hollow structure facilitates the insertion and extraction of lithium-ions due to its expanded specific surface area (Emamdoust and Shayesteh, 2018). Supplementary Figure 1b shows that the TiO_2_ spheres were semi-coated with a thin SnO_2_ shell after incubation for 2 h. When the reaction time was extended to 18 h, thick SnO_2_ nano-flakes were compactly assembled on the surface of TiO_2_ spheres (Supplementary Figure 1c), increasing the risk of pulverization of the active materials during the insertion and extraction of lithium-ions (Gogotsi and Simon, 2011; Li et al., 2017).

**Figure 2:**
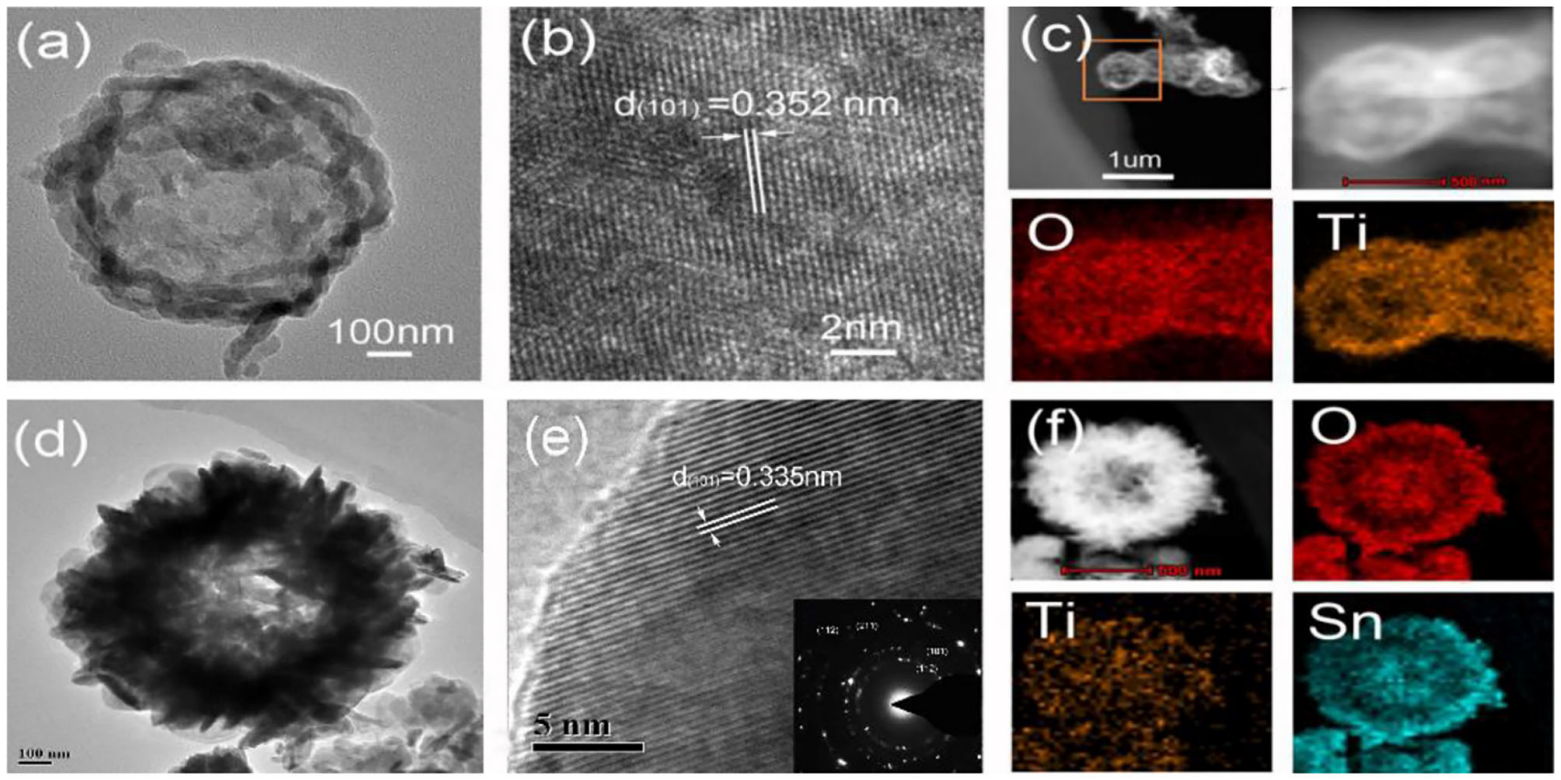
TEM and HRTEM images of **(a,b)** TiO_2_ hollow spheres and **(d,e)** 3D flower-like SnO_2_/TiO_2_ hollow spheres. EDS elemental mapping images of **(c)** TiO_2_ hollow spheres and **(f)** 3D flower-like SnO_2_/TiO_2_ hollow spheres.

Figure 2a presents the TEM images of TiO_2_ hollow spheres. Their centers were in sharp contrast to their edges, showing a distinctly hollow structure. Their relative lattice fringe spacing was 0.352 nm, corresponding to the (101) diffraction planes of rutile TiO_2_ (Figure 2b) (Nguyen et al., 2020). The EDX mapping indicates that Ti and O elements were contained in the TiO_2_ hollow spheres (Figure 2c). After the second hydrothermal reaction, SnO_2_ nano-flakes were vertically assembled on the surface of TiO_2_ hollow spheres (Figure 2d). The flower-like SnO_2_/TiO_2_ products still maintained a distinctly hollow structure. The lattice fringe spacing of flower-like shell was 0.335 nm, matching with the (101) diffraction planes of SnO_2_ (Figure 2e) (Hu et al., 2020). The EDX mapping shows that the flower-like SnO_2_/TiO_2_ hollow spheres were composed of O, Ti and Sn elements (Figure 2f). The Sn element evenly distributed in the flower-like shell of the final hollow spheres, confirming their remarkable core-shell architecture.

**Figure 3:**
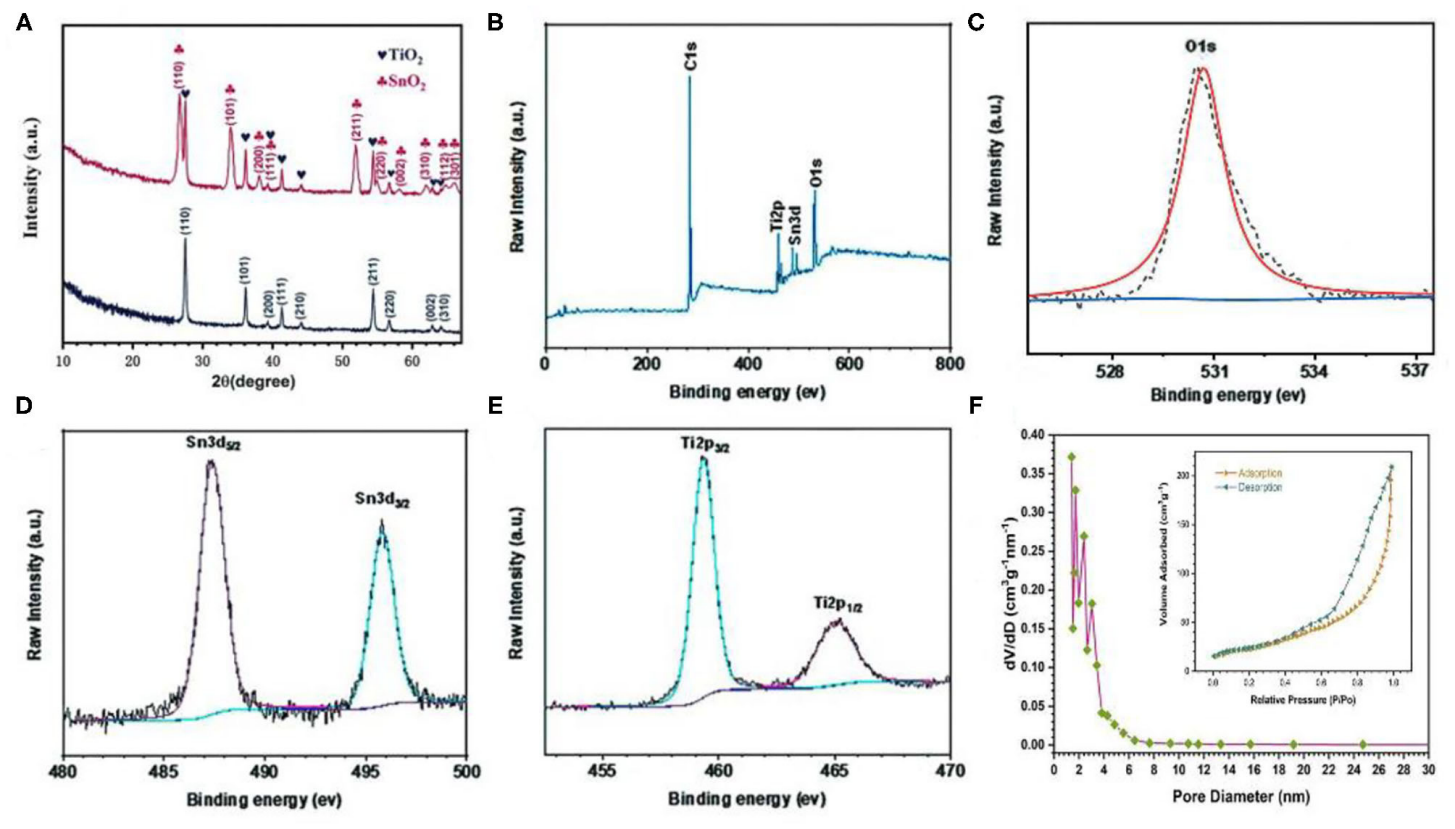
**(A)** XRD pattern of TiO_2_ hollow spheres and 3D flower-like SnO_2_/TiO_2_ hollow spheres, XPS spectra of 3D flower-like SnO_2_/TiO_2_ hollow spheres: **(B)** survey spectra, **(C)** O 1s, **(D)** Sn 3d, **(E)** Ti 2p, **(F)** Nitrogen adsorption/desorption isotherms (inset) and pore size distribution of 3D flower-like SnO_2_/TiO_2_ hollow spheres.

Figure 3A shows the XRD pattern of TiO_2_ and the flower-like SnO_2_/TiO_2_ hollow spheres. The diffraction peaks of the pure TiO_2_ hollow spheres matched well with the standard peaks of rutile crystal phase (JCPDS No. 21-1276). In addition to the diffraction peaks for rutile TiO_2_, the diffraction peaks of the flower-like SnO_2_/TiO_2_ hollow spheres were composed of SnO_2_ (JCPDS NO. 41-1445). The chemical states of the flower-like SnO_2_/TiO_2_ hollow spheres were further investigated by using XPS. The XPS survey spectrum of the flower-like SnO_2_/TiO_2_ hollow spheres contained C, Sn, O, Ti elements (Figure 3B). Figure 3C plots the high-resolution XPS spectrum of O 1s. The peaks at 530.7 in the spectrum of O 1s (Figure 3E) represented oxides formed by O^2−^ composed of Ti and Zn. As shown in Figure 3D, the Sn 3d spectrum had two enhancement peaks at 487.1 and 495.3 eV, corresponding to the Sn^4+^ 3d_5/2_ and Sn^4+^ 3d_3/2_ binding energies of SnO_2_, respectively (Li et al., 2015; Sun et al., 2020). The Ti 2p spectrum in Figure 3E had two characteristic peaks at 459.5 and 465.3eV, which were attributed to Ti^4+^ 2p_3/2_ and Ti^4+^ 2p_1/2_, respectively (Zhang et al., 2015). The XPS results indicate that the obtained products were a mixture of TiO_2_ and SnO_2_, which was consistent with the SEM, TEM and XRD results.

Nitrogen adsorption-desorption measurements further verified the interface advantages of the flower-like SnO_2_/TiO_2_ hollow spheres. Figure 3F shows the nitrogen adsorption-desorption isotherm of the flower-like SnO_2_/TiO_2_ hollow spheres had a type IV isotherm, indicating that the final products were the mesoporous structure. The specific surface area of the flower-like SnO_2_/TiO_2_ hollow spheres was calculated to be about 138.5 m^2^ g^−1^. The pore diameter was mainly distributed between 1 and 8 nm. Their mesoporous structure and larger specific surface area facilitated the insertion and extraction of lithium ions, resulting in a large practical capacity of the final electrode (Liu et al., 2020; Zhong et al., 2020).

**Figure 4:**
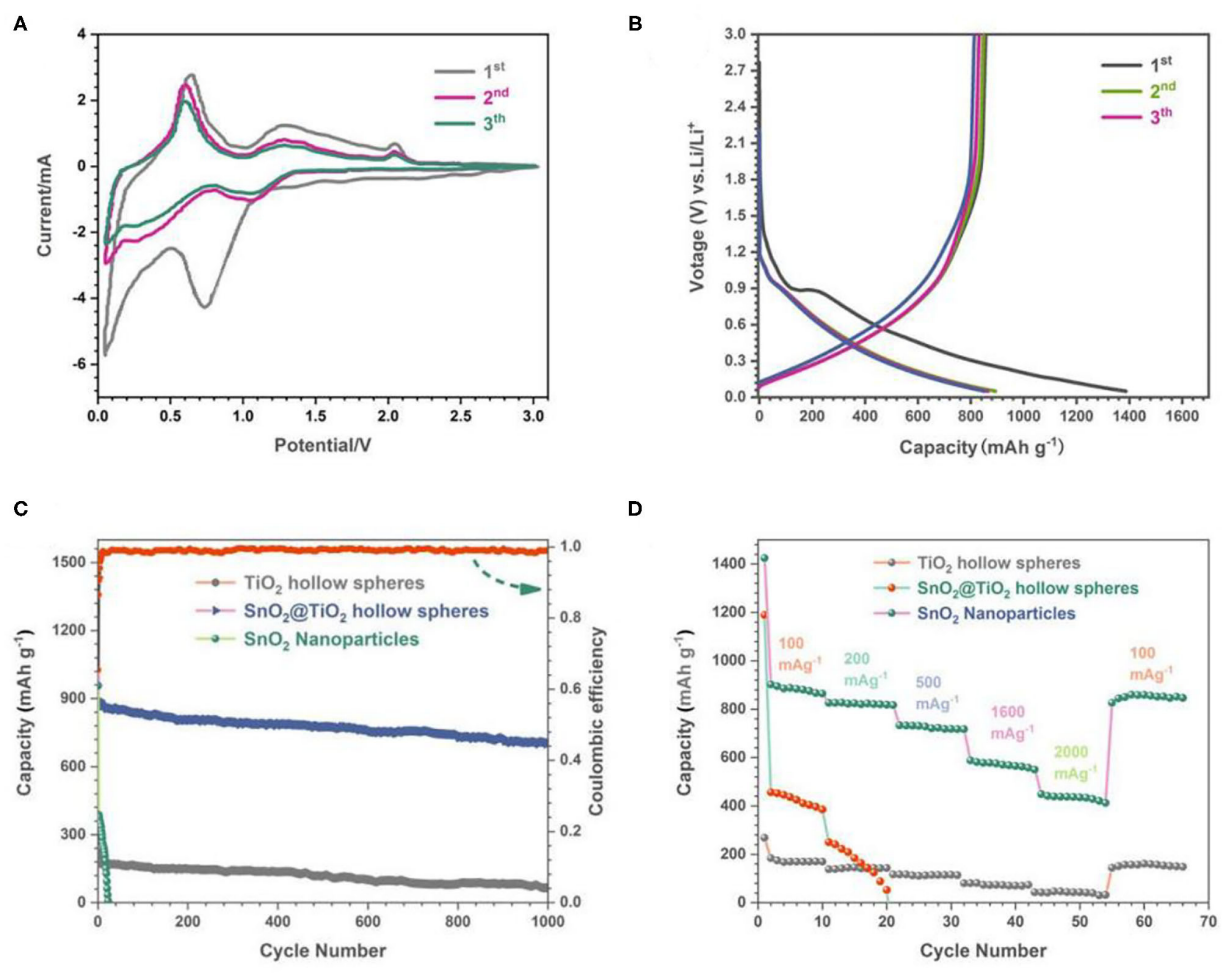
**(A)** CV curves of 3D flower-like SnO_2_/TiO_2_ hollow spheres, **(B)** Charge-discharge voltage profiles of 3D flower-like SnO_2_/TiO_2_ hollow spheres, **(C)** Cycling performance of 3D flower-like SnO_2_/TiO_2_ hollow spheres, TiO_2_ hollow spheres and SnO_2_ nanoparticles. **(D)** Rate performance of 3D flower-like SnO_2_/TiO_2_ hollow spheres, TiO_2_ hollow spheres, and SnO_2_ nanoparticles.

The electrochemical properties of the flower-like SnO_2_/TiO_2_ hollow spheres were tested by using lithium foils as counter and reference electrode. The typical CV curves of 3D flower-like SnO_2_/TiO_2_ hollow spheres were plotted in Figure 4A. In the first cycle, there were two cathodic current peaks at about 0.74 and 1.65 V. The cathodic peak at about 0.74 V corresponded to Sn reduction of SnO_2_ (Wu N. et al., 2018). The cathodic peak around 1.65 V was caused by the SEI layers generated on the surface of the flower-like SnO_2_/TiO_2_ hollow spheres. This peak disappeared in the subsequent cycles given the stable formation of SEI layers. In the anodic process, three obvious peaks appeared at about 0.6, 1.2, and 2.1V, respectively. The oxidation peak at about 0.6 V could be attributed to the dealloying process of Li_x_Sn. The peak appearing around 1.2 V was due to the partially reversible reaction between Sn and SnO_2_. The redox peak at about 2.0V was originated from interaction between lithium ions and TiO_2_ (Jeun et al., 2013; Yuan et al., 2015; Liu Q. et al., 2020). The CV curves overlapped well in the subsequent cycles, indicating that the electrochemical reaction had good reversibility. The above electrochemical reaction mechanism were described as follows:


(1) $$\begin{array}{l} \text{Sn}{\text{O}}_{2}+\;4\text{L}{\text{i}}^{+}\;+\;4{\text{e}}^{-}\;=\text{ Sn }+\;2\text{L}{\text{i}}_{2}\text{O} \end{array}$$

(2) $$\begin{array}{l} \text{Sn }+\text{ xL}{\text{i}}^{+}+\text{ x}{\text{e}}^{-}\;=\;2\text{L}{\text{i}}_{\text{x}}\text{Sn}\left(0\leq \text{x}\leq 4.4\right) \end{array}$$

(3) $$\begin{array}{l} \text{xLi}++\text{ Ti}{\text{O}}_{2}\;+\text{x}{\text{e}}^{-}=2\text{L}{\text{i}}_{\text{x}}\text{Ti}{\text{O}}_{2}\left(0\leq \text{x}\leq 1\right) \end{array}$$

Figure 4B presents the charge-discharge voltage profiles of the flower-like SnO_2_/TiO_2_ hollow spheres at current densities of 200 mA g^−1^. The flower-like SnO_2_/TiO_2_ hollow spheres delivered an initial discharge/charge capacity of 1,372/856 mAh g^−1^. The initial capacity loss was mainly caused by the incomplete conversion reaction and the formation of the SIE layer. The charge-discharge profiles of the second and third curves overlapped well, revealing that the flower-like SnO_2_/TiO_2_ hollow sphere electrodes had good cycling stability. This was consistent with the CV test results.

Figure 4C shows the cycling performance of the flower-like SnO_2_/TiO_2_ hollow spheres, TiO_2_ hollow spheres and SnO_2_ nanoparticles at current densities of 200 mA g^−1^. Compared to the pure TiO_2_ hollow spheres and SnO_2_ nanoparticles, the flower-like SnO_2_/TiO_2_ hollow spheres inherited not only large specific capacity of SnO_2_, but also the good cycling stability of TiO_2_. After 1,000 cycles, their discharge capacity was still maintained at about 720 mAh g^−1^. In contrast, the capacity of pure SnO_2_ nanoparticles decayed rapidly to after 40 cycles. Although the cycling performance of TiO_2_ hollow spheres was stable, their capacity was low. Figure 4D shows the rate performance of the above-mentioned electrodes in the range of 100–2,000 mA g^−1^. The flower-like SnO_2_/TiO_2_ hollow spheres maintained a stable discharge capacity of 887, 826,723, 574, and 486 mAh g^−1^ at a high current density of 100, 200, 500, 1,600, and 2,000 mA g^−1^, respectively. When the current density returned to the 100 mA g^−1^, the stable discharge capacity of the flower-like SnO_2_/TiO_2_ hollow spheres almost also restored. However, the SnO_2_ electrodes almost lost function when the current density increased to 200 mA g^−1^. According to the previous literature (Xia et al., 2016), the large reversible capacity of the flower-like SnO_2_/TiO_2_ hollow spheres was mainly due to their large surface area, which offered more reactive sites for the interface between the active materials and the lithium ions. Compared with the previous literature (Table 1), the less volume change of thin 2D SnO_2_ nano-flakes and TiO_2_ hollow sphere matrix endowed the final electrodes an excellent cycling stability. Furthermore, the mesoporous architecture facilitated the diffusion of lithium ions and further improved the cycling performance of the flower-like SnO_2_/TiO_2_ hollow spheres.

**Table 1:** List of recent work on SnO_2_/TiO_2_ as lithium-ion anodes.

**Anode materials**	**Capacity**	**Current density**	**Cycles**	**References**
3D flower-like SnO_2_/TiO_2_ hollow spheres	720 mA h g^−1^	200 mA g^−1^	1000	This work
Sphere-like SnO_2_/TiO_2_	483 mA h g^−1^	500 mA g^−1^	40	Shen et al., 2021
TiO_2_@SnO_2_ nanotube arrays	700 mA h g^−1^	100 mA g^−1^	100	Liu Q. et al., 2020
TiO_2_@SnO_2_@TiO_2_ triple-shell nanotubes	550 mA h g^−1^	50 mA g^−1^	60	Jean et al., 2017
SnO_2_/TiO_2_ nano-composites	579 mA h g^−1^	0.2 C	100	Ji et al., 2013
TiO_2_(B)@SnO_2_ core-shell hybrid nanowires	463 mA h g^−1^	30 mA g^−1^	50	Mao et al., 2021
TiO_2_@SnO_2_@3DC	576.1 mA h g^−1^	200 mA g^−1^	500	Tian et al., 2018

**Figure 5:**
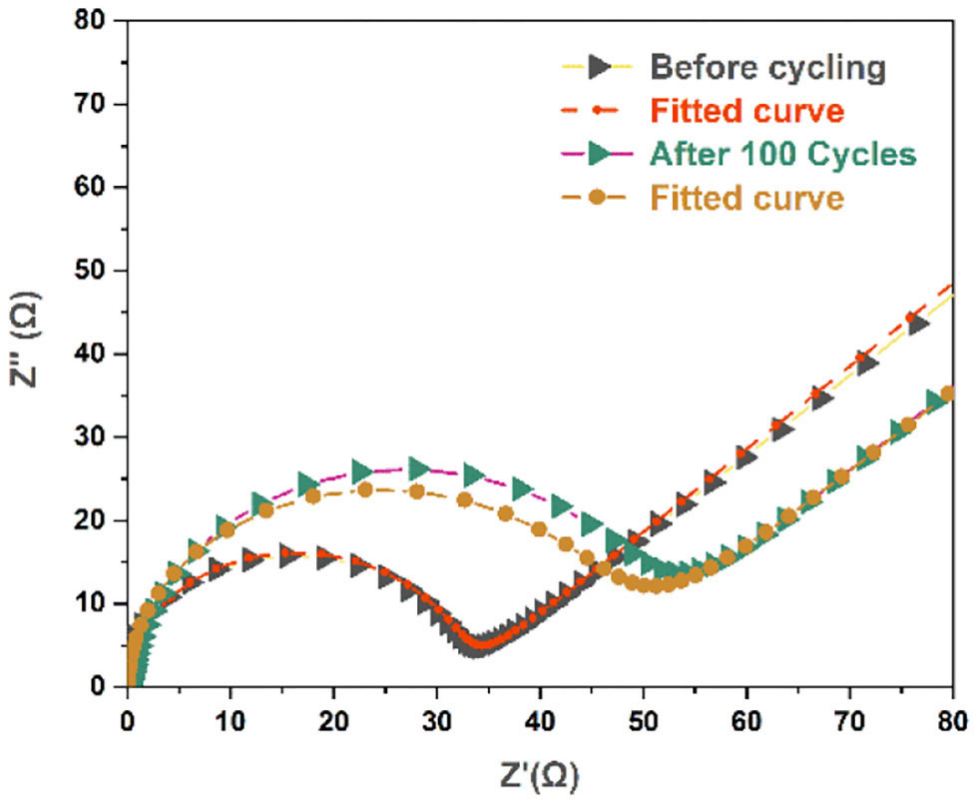
Impedance analysis of 3D flower-like SnO_2_/TiO_2_ hollow spheres.

The superior cycling and rate performance of the flower-like SnO_2_/TiO_2_ hollow spheres was further verified by EIS measurements. Figure 5 and Supplementary Figure 2 illustrate the Nyquist plots of the test specimens before and after cycling. All of the Nyquist plots had a straight line in the low-frequency and a semicircle in the high-frequency region, respectively, presenting the lithium ion diffusion and the charge transfer process. A Randles equivalent circuit was inserted into Figure 5 to simulate the electrochemical system, where Rs was the ohmic resistance, CPE was the double-layer capacitance, R_CT_ was the charge transfer resistance, and W was the Warburg impedance representing the solid-state diffusion of the lithium-ions in the active materials. The semicircle diameter of the 3D flower-like SnO_2_/TiO_2_ hollow spheres and TiO_2_ hollow spheres was significantly smaller than that of SnO_2_ nanoparticles. The charge transfer resistance of the 3D flower-like SnO_2_/TiO_2_ hollow spheres, TiO_2_ hollow spheres and SnO_2_ nanoparticles were about 19.8, 31.6, and 242.5Ω, respectively. This indicates that the 3D flower-like SnO_2_/TiO_2_ hollow spheres also had a lower charge transfer resistance than SnO_2_ nanoparticles. After 100 cycles, the charge transfer resistance of the 3D flower-like SnO_2_/TiO_2_ and TiO_2_ hollow spheres exhibited a less increase, while that of SnO_2_ nanoparticles increased significantly after 15 cycles. The 3D flower-like SnO_2_/TiO_2_ hollow spheres had a good cycling performance, which was attributed to the significant cycling stability of their TiO_2_ hollow sphere matrix and 2D nano-flakes.

## Conclusions

In summary, the flower-like SnO_2_/TiO_2_ hollow spheres were successfully synthesized by facile hydrothermal methods with the help of carbon spheres. Their flower-like shell and mesoporous structure delivered a large specific surface area and excellent ion migration performance. The further electrochemical measurements demonstrated that the flower-like SnO_2_/TiO_2_ hollow spheres exhibited a high specific capacity, low irreversible capacity loss, and superior rate performance. After 1,000 cycles at current densities of 200 mA g^−1^, the capacity of the flower-like SnO_2_/TiO_2_ hollow spheres was still maintained at 720 mAh g^−1^. Their rate capacity reached 486 mAhg^−1^ when the current densities gradually increase to 2,000 mA g^−1^.

## Data Availability Statement

The original contributions presented in the study are included in the article/Supplementary Material, further inquiries can be directed to the corresponding author/s.

## Author Contributions

YW and ZZ conceived and designed the experiments. HZ supervised the research. YZ and XZ helped to synthesize the materials. XX and YW performed the synthesis and characterization, interpreted the data, and wrote the paper with help from ZZ. All authors contributed to the article and approved the submitted version.

## Funding

This work was supported by National Natural Science Foundation of China (Grant No. 71401106), Shanghai Natural Science Foundation (Grant No.14ZR1418700), and Shanghai University of Engineering Science Innovation Fund (Grant No. 18KY0504).

## Conflict of Interest

The authors declare that the research was conducted in the absence of any commercial or financial relationships that could be construed as a potential conflict of interest.

## Publisher's Note

All claims expressed in this article are solely those of the authors and do not necessarily represent those of their affiliated organizations, or those of the publisher, the editors and the reviewers. Any product that may be evaluated in this article, or claim that may be made by its manufacturer, is not guaranteed or endorsed by the publisher.

## References

[B1] Ao S. Zhang Z. Zhang H. Zhao Y. Xu P. Zhou Y.. (2020). Core-etched CC/SnO_2_ nanotube arrays as high-performance anodes for lithium-ion batteries with ionic liquid electrolyte. Front. Mater. 7:63. 10.3389/fmats.2020.00063

[B2] Chen M. Xia X. Yin J. Chen Q. (2015). Construction of Co_3_O_4_ nanotubes as high-performance anode material for lithium ion batteries. Electrochim Acta. 160, 15–21. 10.1016/j.electacta.2015.02.055

[B3] Cheong J. Y. Chang J. H. Kim C. Mweta F. J. Jung J. W. Lee J. Y.. (2017). Revisiting on the effect and role of TiO_2_ layer thickness on SnO2 for enhanced electrochemical performance for lithium-ion batteries. Electrochim Acta. 258, 1140–1148. 10.1016/j.electacta.2017.11.166

[B4] Emamdoust A. Shayesteh S. F. (2018). Surface and electrochemical properties of flower-like Cu-NiO compounds. J. Alloy. Compd. 738, 432–439. 10.1016/j.jallcom.2017.12.144

[B5] Fan L. Lei S. Sari H. M. K. Zhong L. Kakimov A. Wang J.. (2020). Controllable S-Vacancies of monolayered Mo–S nanocrystals for highly harvesting lithium storage. Nano Energy 78:105235. 10.1016/j.nanoen.2020.105235

[B6] Fan X. Liu X. Hu W. Zhong C. Lu J. (2019). Advances in the development of power supplies for the internet of everything. InfoMat. 1, 130–139. 10.1002/inf2.1201625855820

[B7] Gogotsi Y. Simon P. (2011). True performance metrics in electrochemical energy storage. Science 334, 917–918. 10.1126/science.1213003 22096182

[B8] Hu Z. Xu X. Wang X. Yu K. Liang C. (2020). Ultrafine SnO_2_ nanoparticles anchored in the porous corn straw carbon substrate for high-performance Li-ion batteries application. J. Alloy Compd. 835:155446. 10.1016/j.jallcom.2020.155446

[B9] Jean J. H. Kwak H. Kim W. S. Kim H. C. Park K. Y. Kim H.. (2017). TiO_2_@ SnO_2_@TiO_2_ triple-shell nanotube anode for high-performance lithium-ion batteries. J. Solid State Electr. 21, 2365–2371. 10.1007/s10008-017-3584-5

[B10] Jeun H. Park K. Y. Kim D. H. Kim W. S. Kim H. C. Lee B. S.. (2013). SnO_2_@ TiO_2_ double-shell nanotubes for a lithium ion battery anode with excellent high rate cyclability. Nanoscale 5, 8480–8483. 10.1039/c3nr01964k 23897097

[B11] Ji G. Ding B. Ma Y. Lee J. Y. (2013). Nanostructured SnO_2_@TiO_2_ core-shell composites: a high-rate li-ion anode material usable without conductive additives. Energy Technol. 1, 567–572. 10.1002/ente.20130007325855820

[B12] Jiang Z. Liu C. Zhang L. Wei T. Jiang H. Zhou J.. (2019). Ultra-small NiO nanoparticles anchored on nitrogen-doped carbon flowers through strong chemical bonding for high-performance lithium-ion batteries. J. Power Sourc. 441:227182. 10.1016/j.jpowsour.2019.227182

[B13] Lee Y. L. Yi H. W. Kim J. Dong K. K. (2009). Fabricating genetically engineered high-power lithium-ion batteries using multiple virus genes. Science 324, 1051–1055. 10.1126/science.1171541 19342549

[B14] Li P. Deng J. Li Y. Liang W. Wang K. Kang L.. (2014). One-step solution combustion synthesis of Fe2O3/C nano-composites as anode materials for lithium ion batteries. J. Alloy Compd. 590, 318–323. 10.1016/j.jallcom.2013.12.110

[B15] Li R. Xiao W. Miao C. Fang R. Wang Z. Zhang M. (2019). Sphere-like SnO_2_/TiO_2_ composites as high-performance anodes for lithium ion batteries. Ceram Int. 45, 13530–13535. 10.1016/j.ceramint.2019.04.059

[B16] Li S. Ling M. Qiu J. Han J. Zhang S. (2015). Anchoring ultra-fine TiO_2_-SnO_2_ solid solution particles onto graphene by one-pot ball-milling for long-life lithium-ion batteries. J. Mater. Chem. A 3, 9700–9706. 10.1039/C5TA01350J

[B17] Li Y. Wang S. Lei D. He Y.-B. Li B. Kang F. (2017). Acetic acid-induced preparation of anatase TiO_2_ mesocrystals at low temperature for enhanced Li-ion storage. J. Mater. Chem. A 5, 12236–12242. 10.1039/C7TA02361H

[B18] Liu G. Yuan X. Yang Y. Tao J. Chi Y. Hong L.. (2019). Three-dimensional hierarchical wreath-like Co_3_O_4_@ TiO_2_ as an anode for lithium-ion batteries. J. Alloy. Compd. 780, 948–958. 10.1016/j.jallcom.2018.11.242

[B19] Liu Q. Wang L. Zhao K. Yan W. Liu M. Wei D.. (2020). 3D branched rutile TiO2@ rutile SnO_2_ nanorods array heteroarchitectures/carbon cloth with an adjustable band gap to enhance lithium storage reaction kinetics for flexible lithium-ion batteries. Electrochim. Acta 354:136727. 10.1016/j.electacta.2020.136727

[B20] Liu W. Yuan J. Hao Y. Sari H. M. K. Wang J. Kakimov A.. (2020). Heterogeneous structured MoSe_2_-MoO_3_ quantum dots with enhanced sodium/potassium storage. J. Mater. Chem. A 8:23395. 10.1039/D0TA08674F

[B21] Luo W. Wang Y. Wang L. Jiang W. Chou S.-L. Dou S. X.. (2016). Silicon/Mesoporous carbon/crystalline TiO_2_ nanoparticles for highly stable lithium storage. ACS Nano 10, 10524–10532. 10.1021/acsnano.6b06517 27786460

[B22] Mao P. Wang Y. Guo W. Zhang W. He T. Dong S.. (2021). Morphology-controlled synthesis and lithium storage properties of SnO2@ C@ MoS2 hollow nanospheres with petaloid and granular MoS2 nanosheets as the external layer in different solvents. J. Alloy Compd. 850:156745. 10.1016/j.jallcom.2020.156745

[B23] Miao C. Liu M. He Y. B. Qin X. Tang L. Huang B.. (2016). Monodispersed SnO2 nanospheres embedded in framework of graphene and porous carbon as anode for lithium ion batteries. Energy Storage Mater. 3, 98–105 10.1016/j.ensm.2016.01.006

[B24] Nguyen Q. H. Nguyen Q. H. Hur J. (2020). High-Performance ZnTe-TiO_2_-C nanocomposite with half-cell and full-cell applications as promising anode material for Li-Ion batteries. Appl. Surf. Sci. 509:144718. 10.1016/j.apsusc.2019.144718

[B25] Qi X. Zhang H. Zhang Z. Bian Y. Shen A. Xu P. (2018). Subunits controlled synthesis of three-dimensional hierarchical flower-like α-Fe_2_O_3_ hollow spheres as high-performance anodes for lithium ion batteries. Appl. Surf. Sci. 452, 174–180. 10.1016/j.apsusc.2018.04.253

[B26] Scrosati B. Hassoun J. Sun Y. K. (2011). Lithium-ion batteries. A look into the future. Energy Environ. Sci. 4, 3287–3295. 10.1039/c1ee01388b

[B27] Shen H. Xia X. Yan S. Jiao X. Sun D. Lei W.. (2021). SnO_2_/NiFe_2_O_4_/graphene nanocomposites as anode materials for lithium ion batteries. J. Alloy. Compd. 853:157017. 10.1016/j.jallcom.2020.157017

[B28] Sun Q. Kong X. Liu W. Xu B. Hu P. Gao Z.. (2020). Flakes-stacked Sn/SnO_2_/C composite as highly stable anode material for lithium-ion batteries. J. Alloy. Compd. 831:154677. 10.1016/j.jallcom.2020.154677

[B29] Tian H. Liu H. Yang T. Veder J. P. Wang G. Hu M.. (2017). Fabrication of core–shell, yolk–shell and hollow Fe_3_O_4_@ carbon microboxes for high-performance lithium-ion batteries. Mater. Chem. Front. 1, 823–830. 10.1039/C7QM00059F

[B30] Tian Q. Chen Y. Chen F. Zhang W. Chen J. Yang L. (2019). Etching-free template synthesis of double-shelled hollow SiO_2_@ SnO_2_@ C composite as high performance lithium-ion battery anode. J. Alloy. Compd. 809:151793. 10.1016/j.jallcom.2019.151793

[B31] Tian Q. Yan J. Yang L. Chen J. (2018). Fabrication of three-dimensional carbon coating for SnO_2_/TiO_2_ hybrid anode material of lithium-ion batteries. Electrochim. Acta. 282, 38–47. 10.1016/j.electacta.2018.04.044

[B32] Wang Q. Wang D. Wu M Liu B. Chen J. Wang T.. (2011). Porous SnO_2_ nanoflakes with loose-packed structure: morphology conserved transformation from SnS2 precursor and application in lithium ion batteries and gas sensors. J. Phys. Chem. Solids 72, 630–636. 10.1016/j.jpcs.2011.02.004

[B33] Wu B. Xie Y. Meng Y. Qian C. Chen Y. Yuan A.. (2019). Construction of unique heterogeneous cobalt–manganese oxide porous microspheres for the assembly of long-cycle and high-rate lithium ion battery anodes. J. Mater. Chem. A 7, 6149–6160. 10.1039/C8TA09028A

[B34] Wu K. Shi B. Qi L. Mi Y. Zhao B. Yang C.. (2018). SnO_2_ quantum dots@ 3D sulfur-doped reduced graphene oxides as active and durable anode for lithium ion batteries. Electrochim. Acta 291, 24–30. 10.1016/j.electacta.2018.09.086

[B35] Wu N. Du W. Gao X. Zhao L. Liu G. Liu X.. (2018). Hollow SnO_2_ nanospheres with oxygen vacancies entrapped by a N-doped graphene network as robust anode materials for lithium-ion batteries. Nanoscale 10, 11460–11466. 10.1039/C8NR02290A 29888359

[B36] Wu P. Xu X. Zhu Q. Zhu X. Tang Y. Zhou Y.. (2015). Self-assembled graphene-wrapped SnO_2_ nanotubes nanohybrid as a high-performance anode material for lithium-ion batteries. *J. Alloy*. Compd. 626, 234–238. 10.1016/j.jallcom.2014.12.037

[B37] Xia L. Wang S. Liu G. Ding L. Qiao S. (2016). Flexible SnO_2_/N-doped carbon nanofiber films as integrated electrodes for lithium-ion batteries with superior rate capacity and long cycle life. Small 12, 853–859. 10.1002/smll.201503315 26714438

[B38] Xu P. Zhang Z. Zhang H. Shen A. Zhao Y. Zhou Y.. (2020). Binder-free charantia-like metal-oxide core/shell nanotube arrays for high-performance lithium-ion anodes. Front. Chem. 8:159. 10.3389/fchem.2020.00159 32211381 PMC7067744

[B39] Yang Z. Du G. Meng Q. Guo Z Yu X. Chen Z.. (2011). Dispersion of SnO_2_ nanocrystals on TiO_2_ (B) nanowires as anode material for lithium ion battery applications. RSC Adv. 1, 1834–1840. 10.1039/c1ra00500f

[B40] Yuan J. Zhang X. Li H. Wang K. Xie Y. (2015). TiO_2_/SnO_2_ double-shelled hollow spheres-highly efficient photocatalyst for the degradation of rhodamine B. Catal. Commun. 60, 129–133. 10.1016/j.catcom.2014.11.032

[B41] Zhang H. Ren W. Cheng C. (2015). Three-dimensional SnO_2_@ TiO_2_ double-shell nanotubes on carbon cloth as a flexible anode for lithium-ion batteries. Nanotechnology. 26, 274002. 10.1088/0957-4484/26/27/274002 26082042

[B42] Zhang J. Zhang X. Hou Z. Zhang L. Li C. (2019). Uniform SiOx/graphene composite materials for lithium ion battery anodes. J. Alloy. Compd. 809:151798. 10.1016/j.jallcom.2019.151798

[B43] Zhang Z. Xu P. Zhang H. Shen A. Zhao Y. (2019). Flexible three-dimensional titanium-dioxide-based hollow nanoflower arrays for advanced lithium-ion battery anodes. ACS Appl. Energy Mater. 2, 5744–5752. 10.1021/acsaem.9b00869

[B44] Zhong C. Liu B. Ding J. Liu X. Zhong Y. Li Y.. (2020). Decoupling electrolytes towards stable and high-energy rechargeable aqueous zinc–manganese dioxide batteries. Nat. Energy 5, 440–449. 10.1038/s41560-020-0584-y

[B45] Zhu C. Xia X. Liu J. Fan Z. Chao D. Zhang H.. (2014). TiO_2_ nanotube@ SnO_2_ nanoflake core–branch arrays for lithium-ion battery anode. Nano Energy 4, 105–112. 10.1016/j.nanoen.2013.12.018

