# Supplementary material for: TiO2 Hollow Spheres With Flower-Like SnO2 Shell as Anodes for Lithium-Ion Batteries
Source: Front Chem. 2021 Dec 8;9:660309. doi: 10.3389/fchem.2021.660309 (PMC8692286; doi:10.3389/fchem.2021.660309)
Supplement: Supplementary file 1 [file Data_Sheet_1.DOCX]

**TiO_2_ Hollow Spheres with Flower-like SnO_2_ shell as Anodes for Lithium-ion Batteries**

Authors: Ying Weng^a^, Ziying Zhang^*a^, Huizhen Zhang^b^, Yangyang Zhou^a^, Xiaona Zhao^a^, Xingran Xu^a^

^a^School of Materials Engineering, Shanghai University of Engineering Science, Shanghai 201620, China

^b^School of Management, University of Shanghai for Science and Technology, Shanghai 200093, China

*Corresponding Authors: Ziying Zhang, Zaicheng Sun, Shisheng Xiong

E-mail address: [zzying@sues.edu.cn](mailto:zzying@sues.edu.cn)

**
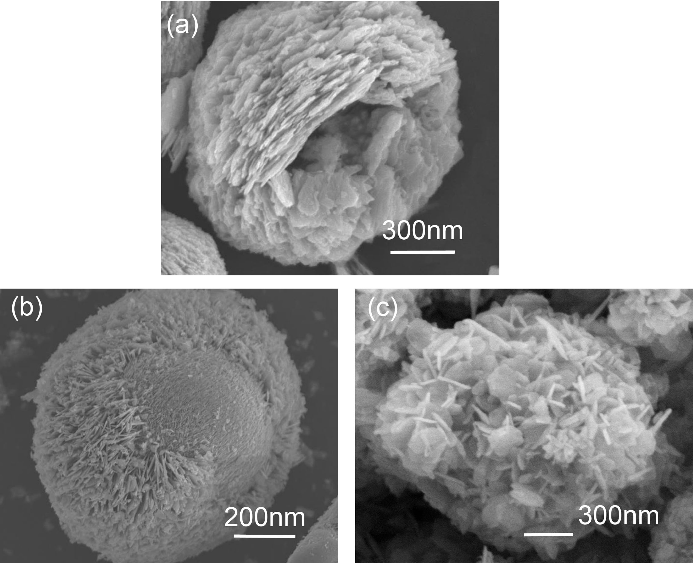
**

Figure S1. HRSEM images of (a) 3D flower-like SnO_2_/TiO_2_ hollow spheres, (b) SnO_2_/TiO_2_ hollow spheres incubated for 2h and (c) SnO_2_/TiO_2_ hollow spheres incubated for 18h.


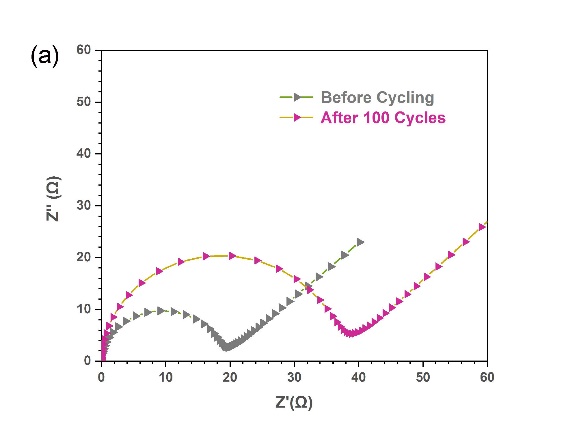

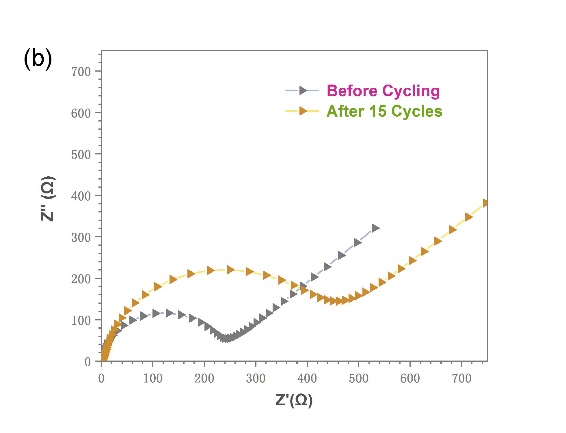


Figure S2. Impedance analysis of (a) TiO_2_ hollow spheres and (b) SnO_2_ nanoparticles.
